# LONGITUDINAL INTERRELATIONSHIP BETWEEN SENSORY LOSS, SOCIAL SUPPORT, LONELINESS, AND COGNITION

**DOI:** 10.1093/geroni/igz038.3015

**Published:** 2019-11-08

**Authors:** Shaoqing Ge, Wei Pan, Bei Wu, Brenda Plassman, Eleanor S McConnell

**Affiliations:** 1 Duke University School of Nursing, North Carolina, United States; 2 Duke University School of Nursing, Durham, North Carolina, United States; 3 NYU Rory Meyers College of Nursing, New York, New York, United States; 4 Duke Department of Neurology, Durham, North Carolina, United States

## Abstract

This study aims to understand the roles that psychosocial factors play on the longitudinal associations between sensory (including hearing and vision) loss and cognitive decline. Specifically, we hypothesized that (1) loneliness mediates the associations between sensory loss and cognitive decline; and (2) social support moderates the associations between sensory loss and cognitive decline. We used longitudinal parallel process (LPP) modeling with data from the Health and Retirement Study (HRS) and the Aging, Demographics, and Memory study (ADAMS). Age variable centered at its mean age of 82. In the most parsimonious model, loneliness fully mediated the associations between vision loss and the average cognitive status at age 82 (p < .05). Social support moderated the associations between vision loss and the average cognitive status at age 82 (β= .14, p < .05). No moderation or mediation effect was found for the psychosocial factors on the associations between hearing loss and cognition.

